# Fasting glucose-to-HDL cholesterol ratio and risk of MASLD in nonobese Chinese adults: A retrospective cohort study

**DOI:** 10.1097/MD.0000000000051474

**Published:** 2026-11-06

**Authors:** Jian Luo, Zhijie Zhuang, Yingzhu Xiong, Haifeng Zhou, Fang Yi, Danfeng Gong, Liqun Nie, Xueyan Wu

**Affiliations:** a Department of Endocrinology, Changde Hospital, Xiangya School of Medicine, Central South University (The First People’s Hospital of Changde City), Changde, China; b Department of Gastroenterology, Changde Hospital, Xiangya School of Medicine, Central South University (The First People’s Hospital of Changde City), Changde, China; c Department of Neurophysiology, Changde Hospital, Xiangya School of Medicine, Central South University (The First People’s Hospital of Changde City), Changde, China.

**Keywords:** FHR, hepatic steatosis, lipid metabolism, MASLD, metabolic biomarkers, nonobese adults

## Abstract

Metabolic dysfunction-associated steatotic liver disease (MASLD) is increasingly recognized in nonobese individuals; however, reliable markers for early risk identification remain limited. The fasting glucose-to-high-density lipoprotein cholesterol (FHR) ratio, which reflects integrated glucolipid metabolism, has emerged as a potential indicator. This study aimed to evaluate the association between FHR and the incidence of MASLD in nonobese adults. This retrospective cohort study included 16,173 nonobese Chinese adults (body mass index < 25 kg/m^2^) from the Wenzhou Health Examination Cohort. Participants without MASLD at baseline were categorized into quartiles according to their FHR levels. Incident MASLD was diagnosed using abdominal ultrasonography during follow-up. Cox proportional hazards models were used to estimate hazard ratios (HRs) and 95% confidence intervals (CIs) after adjusting for demographic, clinical, and metabolic factors. Dose–response relationships were assessed using restricted cubic spline models, and receiver operating characteristic (ROC) analysis was performed to evaluate predictive performance. During follow-up, 2322 participants developed MASLD. Higher FHR levels were significantly associated with an increased risk of MASLD. In the fully adjusted model, each 1-unit increase in FHR was associated with a 22% higher risk of MASLD (HR, 1.22; 95% CI: 1.17–1.27, *P* < .001). Compared to the lowest quartile, the highest quartile showed a significantly elevated risk (HR: 2.10, 95% CI: 1.78–2.48; *P* for trend < 0.001). A positive dose–response relationship was also observed. ROC analysis showed that FHR had a moderate predictive ability (AUC, 0.701; 95% CI, 0.690 to 0.713), with an optimal cutoff value of 3.87. Elevated FHR was independently associated with an increased risk of MASLD in nonobese Chinese adults. Thus, FHR may serve as a simple and cost-effective biomarker for early risk stratification in this population.

## 1. Introduction

Metabolic dysfunction-associated steatotic liver disease (MASLD), formerly known as nonalcoholic fatty liver disease (NAFLD),^[1]^ has emerged as the leading cause of chronic liver disease globally, with a prevalence that continues to rise at an alarming rate.^[2,3]^ As a major contributor to the global burden of chronic non-communicable diseases, MASLD has been increasingly recognized as a pressing public health concern. It not only progresses to end-stage hepatic sequelae, including cirrhosis and hepatocellular carcinoma,^[4,5]^ but has also been established as an independent risk factor for a spectrum of systemic comorbidities, including type 2 diabetes mellitus, cardiovascular disease, and chronic kidney disease.^[6–8]^ Consequently, MASLD poses a substantial public health and economic burden worldwide.^[9]^ A recent international expert consensus redefined the diagnostic criteria for this condition, shifting from the exclusionary definition of NAFLD to a positive association of the disease with metabolic dysfunction. This paradigm shift, embodied in the new nomenclature of MASLD, more accurately reflects the core pathophysiological mechanisms underlying the disease.^[1,10]^

Although MASLD has traditionally been considered a hepatic manifestation of obesity, a clinically significant subtype – “non-obese” or “lean” MASLD – is gaining increasing scientific attention.^[11]^ Studies have shown that among the MASLD patient population, 19.2% are lean and 40.8% are nonobese. The prevalence of nonobese MASLD in the general population exhibits regional variations, with proportions exceeding 50% in some countries.^[3]^ Compared to their obese counterparts, patients with nonobese MASLD may exhibit distinct genetic susceptibilities and pathophysiological features, such as a higher proportion of visceral adiposity, more pronounced insulin resistance, and a unique atherogenic lipid profile.^[12]^ This hidden subgroup remains underrecognized in clinical and community settings, leading to delayed diagnosis and missed opportunities for early intervention. From a public health perspective, the identification of inexpensive, noninvasive, and easy-to-implement biomarkers is essential for population-level screening and risk stratification, particularly in resource-limited settings where imaging-based screening is impractical.

Composite indices that integrate information on glucose and lipid metabolism have shown great promise in the search for such biomarkers. Among these, the fasting glucose-to-high-density lipoprotein cholesterol (HDL) cholesterol ratio (FHR) has emerged as an attractive candidate because it theoretically captures 2 core pillars of metabolic dysregulation: insulin resistance and atherogenic dyslipidemia.^[13-15]^ Several recent cross-sectional studies have provided preliminary evidence for this association. For instance, a large-scale analysis of the US National Health and Nutrition Examination Survey (NHANES) confirmed that an elevated FHR was significantly associated with the prevalence of MASLD in American adults.^[16]^ A similar association was observed in a Japanese cohort.^[17]^ These findings align with a growing body of evidence on other cardiometabolic indices, such as the triglyceride-glucose (TyG) index and remnant cholesterol, collectively highlighting the critical role of assessing glucolipid metabolic disturbances in MASLD risk stratification.^[18,19]^

However, despite these encouraging preliminary findings, the clinical application of FHR as a predictive metabolic marker remains limited by several important gaps in knowledge. First, most of the existing evidence has been derived from Western populations, whereas ethnic differences in genetic background, dietary patterns, and metabolic characteristics may substantially influence lipid metabolism and susceptibility to MASLD. Second, the predominance of cross-sectional study designs in previous research prevents an accurate evaluation of temporal relationships between FHR and disease onset, thereby limiting the confirmation of its predictive value rather than its purely diagnostic value. This methodological limitation may also contribute to the inconsistent FHR cutoff values reported across studies, which likely reflect current metabolic conditions instead of future disease risk. Finally, the longitudinal association between FHR and incident MASLD in nonobese individuals remains largely unexplored, although this subgroup is known to have distinct metabolic and hepatic features compared to those with obesity.

The Wenzhou Health Examination Cohort has previously been used to investigate the association between low-density lipoprotein cholesterol (LDL-C) and incident NAFLD in nonobese adults.^[20]^ Using the same publicly available cohort data, we conducted a secondary analysis of this retrospective cohort to examine a different metabolic indicator: the fasting plasma glucose to HDL-C ratio (FHR). By combining fasting plasma glucose and HDL-C in a single measure, FHR may capture aspects of both glucose and lipid metabolism. This study aimed to evaluate the association between baseline FHR and subsequent MASLD detection among nonobese Chinese adults and to assess whether this association persisted after adjustment for potential confounders.

## 2. Materials and methods

### 2.1. Study design and data source

This longitudinal retrospective cohort study is a secondary analysis of publicly available data from the Wenzhou Health Examination Cohort in China, originally reported by Sun et al.^[20]^ The original data were deposited in the “Dryad” Digital Repository (DOI: 10.5061/dryad.1n6c4m4) under the Creative Commons Attribution 4.0 International License. The present analysis uses the same source cohort and retains the original eligibility restrictions, including body mass index (BMI) < 25 kg/m^2^ and LDL-C ≤ 3.12 mmol/L. Thus, it does not represent a newly recruited or independent cohort. Whereas Sun et al investigated the association between LDL-C levels within the normal range and incident NAFLD, the present analysis examines the association between baseline FHR and subsequent MASLD detection.

Original cohort data were collected from adults who underwent annual health examinations at the Health Management Center in Wenzhou City, Zhejiang Province, China, between January 2010 and December 2014. The study protocol complied with the principles of the Declaration of Helsinki. The original study was approved by the Ethics Committee of the First Affiliated Hospital of Wenzhou Medical University.^[20]^

### 2.2. Study population

Participants were selected from a longitudinal cohort that initially enrolled 33,153 individuals who underwent annual health examinations at the Medical Center of Wenzhou People’s Hospital between January 2010 and December 2014. First, we excluded participants with fatty liver at baseline. Subsequently, to ensure the homogeneity of the study population and reduce confounding factors, we further excluded individuals who met one or more of the following criteria: alcohol abuse (n = 3315); use of antihypertensive, antidiabetic, or lipid-lowering agents (n = 2272); other known causes of chronic liver disease, such as viral hepatitis (n = 1492); baseline low-density lipoprotein cholesterol (LDL-C) level > 3.12 mmol/L (n = 3320); baseline body mass index (BMI) ≥ 25 kg/m^2^ (n = 4260); or those who were lost to follow-up (n = 2321). After this rigorous screening process, 16,173 eligible nonobese individuals were included in the final longitudinal analysis (Fig. 1).

**Figure 1.**
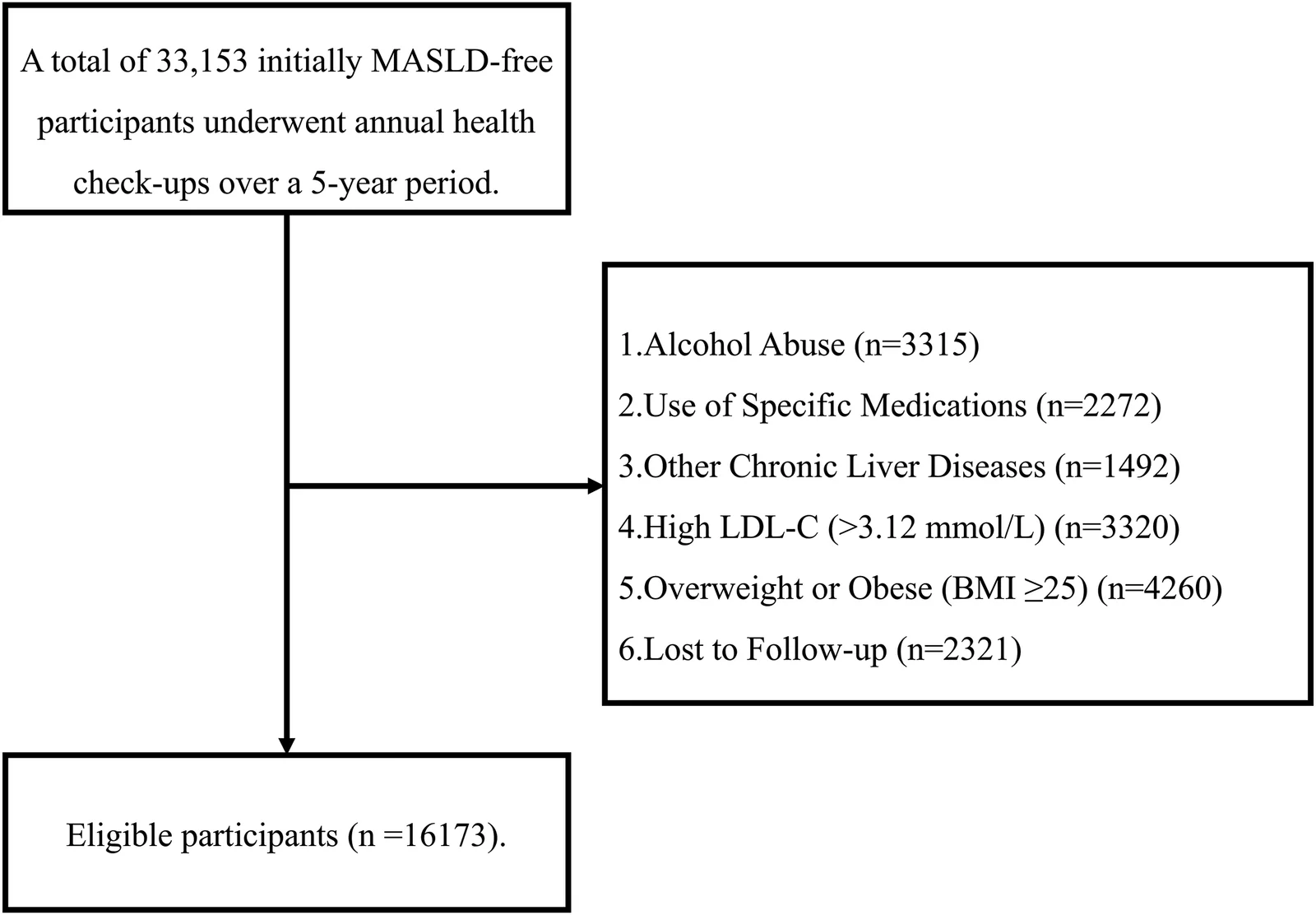
Flow chart. BMI = body mass index, LDL-C = low-density lipoprotein cholesterol, MASLD = metabolic dysfunction-associated steatotic liver disease, n = number.

### 2.3. Data collection and clinical measurements

Baseline data for all participants were collected during the first health examination using a standardized protocol.

#### 2.3.1. Questionnaire and anthropometric measurements

A standardized questionnaire was administered to collect the participants’ demographic information and medical history in the original cohort. Although smoking status and alcohol consumption were collected in the original cohort, these variables were not included in the shared dataset analyzed in the present study and, therefore, could not be adjusted for in the statistical models. Height and weight were measured to the nearest 0.1 cm and 0.1 kg, respectively, by trained professionals. Body Mass Index (BMI) was calculated as weight (kg) divided by height in meters squared (m^2^). After the participants had rested in a seated position for at least 5 minutes, sitting blood pressure was measured 3 times using an electronic sphygmomanometer. The average of 3 readings was used for both systolic (SBP) and diastolic blood pressure (DBP).

#### 2.3.2. Laboratory and biochemical assays

Venous blood samples were drawn from the antecubital vein after overnight fasting for at least 8 hours. The serum was separated by centrifugation within 2 hours of collection. All biochemical parameters were measured using an automated analyzer (Hitachi 7600, Tokyo, Japan). These parameters included fasting plasma glucose (FPG); lipid profiles, including total cholesterol (TC), triglycerides (TG), high-density lipoprotein cholesterol (HDL-C), and low-density lipoprotein cholesterol (LDL-C); liver function indicators, namely alanine aminotransferase (ALT), aspartate aminotransferase (AST), gamma-glutamyl transferase (GGT), alkaline phosphatase (ALP), total protein (TP), and albumin (ALB); and renal function markers, including blood urea nitrogen (BUN), creatinine (Cr), and uric acid (UA). All assays were performed under stringent quality control at the Central Laboratory of the First Affiliated Hospital of the Wenzhou Medical University.

### 2.4. Definition of variables and outcome assessment

#### 2.4.1. Exposure variable

The primary exposure variable in this study was FHR, which was calculated using the formula: FHR = FPG (mmol/L)/HDL-C (mmol/L). For statistical analysis, FHR was treated both as a continuous variable and as a categorical variable and categorized into quartiles (Q1–Q4).

#### 2.4.2. Outcome event

The primary outcome of this study was the incidence of MASLD. Although the original data were collected using the diagnosis of NAFLD, this study adopts the more precise nomenclature of MASLD in accordance with the 2023 expert consensus update.^[21]^ The diagnosis of MASLD was based on the results of abdominal ultrasonography, which were independently evaluated by 2 experienced radiologists. The sonographic criteria for diagnosing hepatic steatosis included diffuse hyperechogenicity of the liver, far-field echo attenuation, obscuration of intrahepatic ductal structures, and increased hepatorenal echo contrast.

The participants underwent annual follow-up health examinations after enrollment. Incident MASLD was defined as the first diagnosis of hepatic steatosis on ultrasonography during the follow-up period. The follow-up time was calculated from the date of the baseline examination to the date of the first MASLD diagnosis, the date of the last follow-up visit, or the study cutoff date, whichever came first.

### 2.5. Patients and public involvement

Patients and the public were not involved in the design, implementation, reporting, or any other aspects of the study.

### 2.6. Statistical analysis

All statistical analyses were performed using R software version 4.2.0 (R Foundation for Statistical Computing, Vienna, Austria) and EmpowerStats version 4.0 (X&Y Solutions, Inc., Boston). A 2-sided *P*-value < .05 was considered statistically significant for all tests.

#### 2.6.1. Missing data handling

Prior to the analysis, we first assessed the completeness of the data for all variables. It was confirmed that none of the variables had a missingness proportion exceeding 25%. For covariates with missing values (all with <25% missingness), we employed multiple imputation by chained equations (MICE) using the random forest algorithm to generate 5 completely imputed datasets. All subsequent analyses were performed independently for each of the 5 datasets, and the final results were pooled according to Rubin’s rules. To validate the robustness of the imputation, we conducted a sensitivity analysis by comparing the results from the complete-case analysis with those from multiple imputation datasets. The conclusions were highly consistent, indicating that the imputation method did not introduce significant bias (Table S1, Supplemental Digital Content 1).

#### 2.6.2. Baseline characteristics

The baseline characteristics of the study population are presented according to FHR quartiles. Continuous variables are expressed as mean ± standard deviation (SD) or median (interquartile range, IQR) and compared using ANOVA or the Kruskal–Wallis test, as appropriate. Categorical variables are presented as frequencies (percentages) and were compared using the chi-squared test.

#### 2.6.3. Association and dose–response analysis

To investigate the association between FHR and the risk of incident MASLD, we applied multivariable Cox proportional hazard regression models to calculate hazard ratios (HRs) and their 95% confidence intervals (CIs). These models were applied to pooled imputed datasets. FHR was analyzed both as a continuous variable and as a categorical variable (quartiles), and the *P* for trend was calculated across the quartiles. We employed a restricted cubic spline (RCS) model with 4 knots to flexibly model and visualize the potentially nonlinear dose–response relationship between FHR and MASLD risk.

#### 2.6.4. Survival and subgroup analyses

The Kaplan–Meier method was used to plot the cumulative incidence curves for different FHR groups, and the log-rank test was used to assess the differences between groups. To examine the consistency of the association, we conducted prespecified subgroup analyses and tested for interactions by including a multiplicative interaction term in the Cox models.

#### 2.6.5. Predictive performance

Finally, we used receiver operating characteristic (ROC) curves to evaluate, and the DeLong test to compare, the predictive performance and area under the curve (AUC) of FHR, HDL-C, and FPG for incident MASLD.

## 3. Results

### 3.1. Baseline characteristics and incidence of MASLD

A total of 16,173 eligible nonobese participants were included in the study and followed for a median of 34.6 months. The participants were stratified into quartiles based on baseline FHR levels: Q1 (n = 4043), Q2 (n = 3934), Q3 (n = 4152), and Q4 (n = 4044). Baseline demographic and clinical characteristics of the study population are shown in Table 1. The mean (±SD) FHR values for the quartiles were 2.55 ± 0.29, 3.23 ± 0.17, 3.86 ± 0.22, and 4.82 ± 0.60, respectively. Across the FHR quartiles, there was a significant increasing trend in ALB, ALP, globulin (GLB), TP, LDL-C, TG, BUN, Cr, UA, FPG, height, weight, BMI, DBP, and SBP. Conversely, higher FHR levels were associated with lower HDL-C and TC levels (all *P *< .001).

**Table 1:** Characteristics of the subjects.

FHR quartile	Q1 (2.55 ± 0.29)	Q2 (3.23 ± 0.17)	Q3 (3.86 ± 0.22)	Q4 (4.82 ± 0.60)	*P*-value
N (%)	4043	3934	4152	4044	
Sex					<.001
Female	2040 (50.46%)	1921 (48.83%)	1957 (47.13%)	1772 (43.82%)	
Male	2003 (49.54%)	2013 (51.17%)	2195 (52.87%)	2272 (56.18%)	
Age, yr	42.47 ± 14.82	42.68 ± 14.53	43.41 ± 15.05	44.33 ± 15.35	<.001
MASLD					<.001
No	3838 (94.93%)	3572 (90.80%)	3447 (83.02%)	2994 (74.04%)	
Yes	205 (5.07%)	362 (9.20%)	705 (16.98%)	1050 (25.96%)	
ALP, U/L	68.00 ± 17.02	70.21 ± 18.52	73.52 ± 22.87	75.24 ± 20.74	<.001
GGT, U/L	22.00 (16.00–22.00)	22.00 (17.00–25.00)	22.00 (19.00–29.00)	22.00 (20.00–34.00)	<.001
ALT, U/L	16.00 (13.00–16.00)	16.00 (13.00–19.00)	16.00 (14.00–21.00)	17.00 (15.00–24.00)	<.001
AST, U/L	21.00 (19.00–22.00)	21.00 (19.00–23.00)	21.00 (19.00–24.00)	21.00 (20.00–25.00)	<.001
TP, g/L	73.56 ± 4.11	73.77 ± 3.85	73.95 ± 3.99	74.23 ± 4.04	<.001
ALB, g/L	44.40 ± 2.72	44.39 ± 2.57	44.35 ± 2.61	44.49 ± 2.46	.092
GLB, g/L	29.18 ± 3.61	29.39 ± 3.58	29.60 ± 3.72	29.75 ± 3.85	<.001
BUN, mmol/L	4.47 ± 1.36	4.49 ± 1.31	4.55 ± 1.28	4.75 ± 1.49	<.001
CR, µmo/L	73.21 ± 19.65	77.78 ± 19.94	79.86 ± 26.41	83.01 ± 33.13	<.001
UA, mmol/L	248.47 ± 75.82	269.61 ± 85.60	288.61 ± 83.57	312.03 ± 85.34	<.001
FPG, mmol/L	4.83 ± 0.40	5.00 ± 0.42	5.30 ± 1.11	5.44 ± 0.79	<.001
TC, mmol/L	4.84 ± 0.71	4.63 ± 0.70	4.57 ± 0.76	4.46 ± 0.75	<.001
TG, mmol/L	0.94 ± 0.40	1.10 ± 0.50	1.40 ± 1.03	1.77 ± 1.21	<.001
HDL-C, mmol/L	1.92 ± 0.26	1.55 ± 0.15	1.30 ± 0.17	1.08 ± 0.17	<.001
LDL-C, mmol/L	2.19 ± 0.47	2.28 ± 0.46	2.31 ± 0.46	2.27 ± 0.46	<.001
Height, m	1.62 ± 0.07	1.64 ± 0.08	1.65 ± 0.08	1.66 ± 0.08	<.001
Weight, kg	54.49 ± 7.56	57.03 ± 8.20	59.16 ± 8.38	61.60 ± 8.35	<.001
BMI, kg/m^2^	20.61 ± 2.00	21.15 ± 2.01	21.59 ± 1.98	22.16 ± 1.88	<.001
SBP, mm Hg	116.72 ± 15.64	118.66 ± 16.22	121.90 ± 16.78	125.54 ± 16.79	<.001
DBP, mm Hg	70.74 ± 9.73	71.84 ± 10.17	73.39 ± 10.43	75.22 ± 10.48	<.001

Values are n (%) or mean (standard deviation) or median (Q1–Q3).

ALB = albumin, ALP = alkaline phosphatase, ALT = alanine aminotransferase, AST = aspartate aminotransferase, BMI = body mass index, BUN = blood urea nitrogen, Cr = creatinine, DBP = diastolic blood pressure, FHR = fasting plasma glucose to high-density lipoprotein cholesterol ratio, FPG = fasting plasma glucose, GGT = gamma-glutamyl transferase, GLB = globulin, HDL-C = high-density lipoprotein cholesterol, LDL-C = low-density lipoprotein cholesterol, MASLD = Metabolic Dysfunction-Associated Steatotic Liver Disease, SBP = systolic blood pressure, TC = total cholesterol, TG = triglyceride, TP = total protein, UA = uric acid.

During the follow-up period, 2322 incident cases of MASLD were recorded, corresponding to an overall incidence rate of 14.4%. A significant difference in the incidence of MASLD was observed across FHR quartiles, with rates of 5.07% in Q1, 9.20% in Q2, 16.98% in Q3, and 25.96% in Q4 (Table 1).

### 3.2. Association between FHR and the risk of incident MASLD

The associations between FHR and incident MASLD in the crude, demographic-adjusted, and fully adjusted Cox proportional hazards models are presented in Table 2. FHR remained significantly and positively associated with incident MASLD after full adjustment. When analyzed as a continuous variable, each 1-unit increase in FHR was associated with a 22% higher hazard of incident MASLD in the fully adjusted model (HR, 1.22; 95% CI: 1.17–1.27, *P* < .0001). When FHR was analyzed as a categorical variable, with the lowest quartile (Q1) as the reference group, the risk of developing MASLD was significantly higher in quartiles Q2, Q3, and Q4. In the fully adjusted model, the corresponding HRs (95% CI) for Q2, Q3, and Q4 were 1.36 (1.14–1.62), 1.80 (1.52–2.12), and 2.10 (1.78–2.48), respectively (all *P* < .001). The trend across the FHR quartiles revealed a significant linear trend (*P* < .0001; Table 2).

**Table 2:** Association between FHR and MASLD in different models.

Exposure	MASLD, HR (95%CI)		
	Crude model	Model 1	Model 2
FHR	1.59 (1.54–1.65) **<**.0001	1.58 (1.53–1.64) **<**.0001	1.22 (1.17–1.27) **<** .0001
FHR quartile			
Q1	1.0	1.0	1.0
Q2	1.94 (1.63–2.30) **<**.0001	1.93 (1.63–2.30) **<**.0001	1.36 (1.14–1.62) .0006
Q3	3.40 (2.91–3.98) **<**.0001	3.38 (2.89–3.95) **<**.0001	1.80 (1.52–2.12) **<** .0001
Q4	4.96 (4.26–5.76) **<**.0001	4.89 (4.21–5.69) **<**.0001	2.10 (1.78–2.48) **<**.0001
*P* for trend	<.0001	<.0001	<.0001

Crude model adjusted for none; model 1 adjusted for: sex; age; model 2 adjusted for: sex; age; TP; ALB; GLB; ALP; GGT; ALT; AST; BUN; CR; UA; TC1; TG; LDL; SBP; DBP; BMI.

Intermediate adjustment models were also evaluated and showed consistent trends in the association between FHR and incident MASLD.

ALB = albumin, ALP = alkaline phosphatase, ALT = alanine aminotransferase, AST = aspartate aminotransferase, BMI = body mass index, BUN = blood urea nitrogen, Cr = creatinine, DBP = diastolic blood pressure, FHR = fasting plasma glucose to high-density lipoprotein cholesterol ratio, FPG = fasting plasma glucose, GGT = gamma-glutamyl transferase, GLB = globulin, HDL-C = high-density lipoprotein cholesterol, HR = hazard ratio, LDL-C = low-density lipoprotein cholesterol, MASLD = Metabolic Dysfunction-Associated Steatotic Liver Disease, SBP = systolic blood pressure, TC = total cholesterol, TG = triglyceride, TP = total protein, UA = uric acid.

### 3.3. Dose–response relationship and cumulative incidence of MASLD by FHR

Restricted cubic spline (RCS) analysis revealed a positive and nonlinear dose–response relationship between FHR levels and log relative risk (Log RR) of incident MASLD (Fig. 2A). Risk consistently increased with increasing FHR, with the curve exhibiting a particularly steep slope at FHR values below 3.5 (Table S2, Supplemental Digital Content 2). Stratified analyses by sex showed that the dose–response curves had a similar shape for both males and females, with a steeper slope observed below an FHR of 3.5 in both sexes (Fig. 2B and Table S2, Supplemental Digital Content 2).

**Figure 2.**
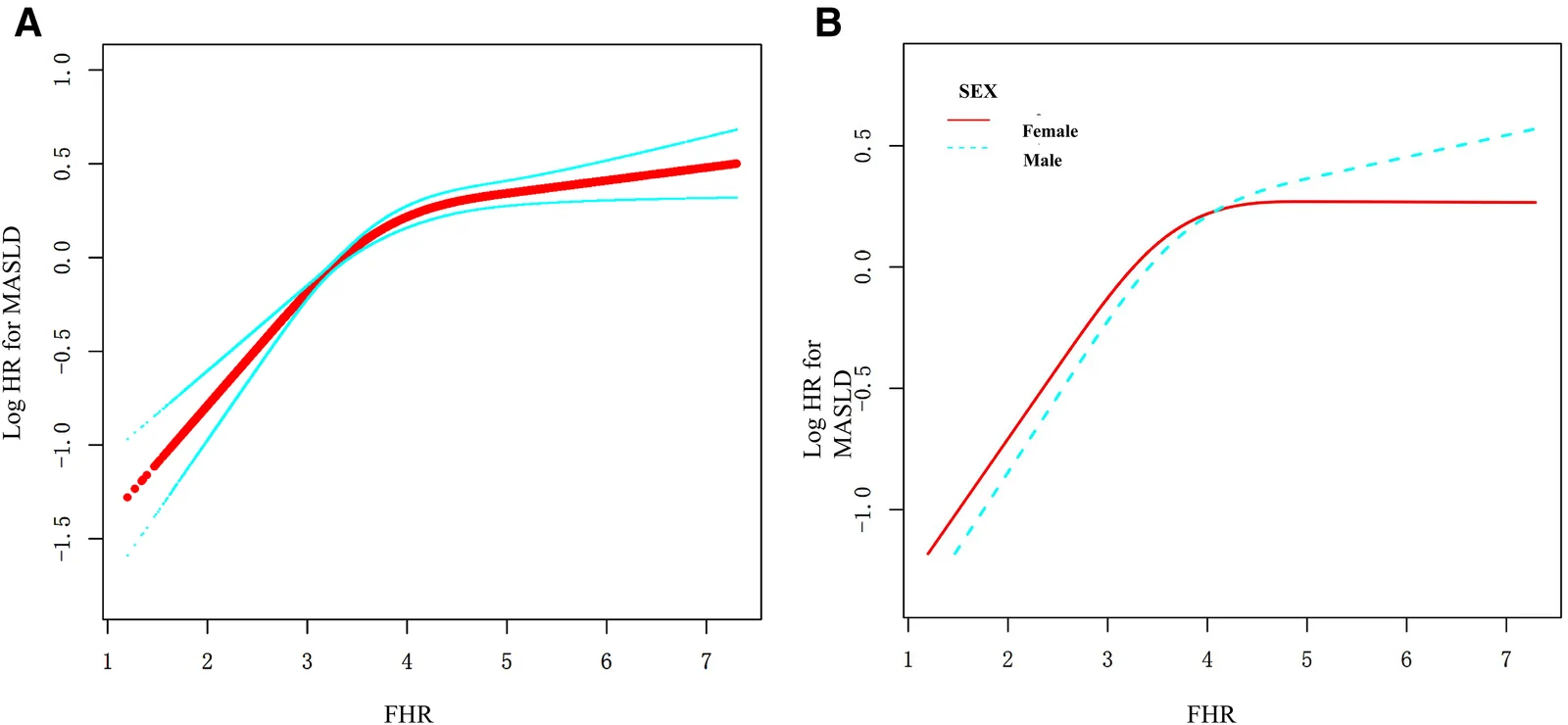
Dose–response relationship between FHR and MASLD risk. (A) Nonlinear association between FHR and log relative risk for MASLD in the overall population. (B) Sex-stratified analysis of the FHR-MASLD association (red line: female; cyan dashed line: male). FHR = fasting plasma glucose to high-density lipoprotein cholesterol ratio, HR = hazard ratios, MASLD = metabolic dysfunction-associated steatotic liver disease.

Kaplan–Meier survival curves visually demonstrated the cumulative incidence of MASLD across the different FHR groups (Fig. 3). During a median follow-up of 34.6 months, the groups with higher FHR levels had a progressively lower probability of remaining free of MASLD. The 4 survival curves were significantly different from each other (log-rank test, *P* < .0001).

**Figure 3.**
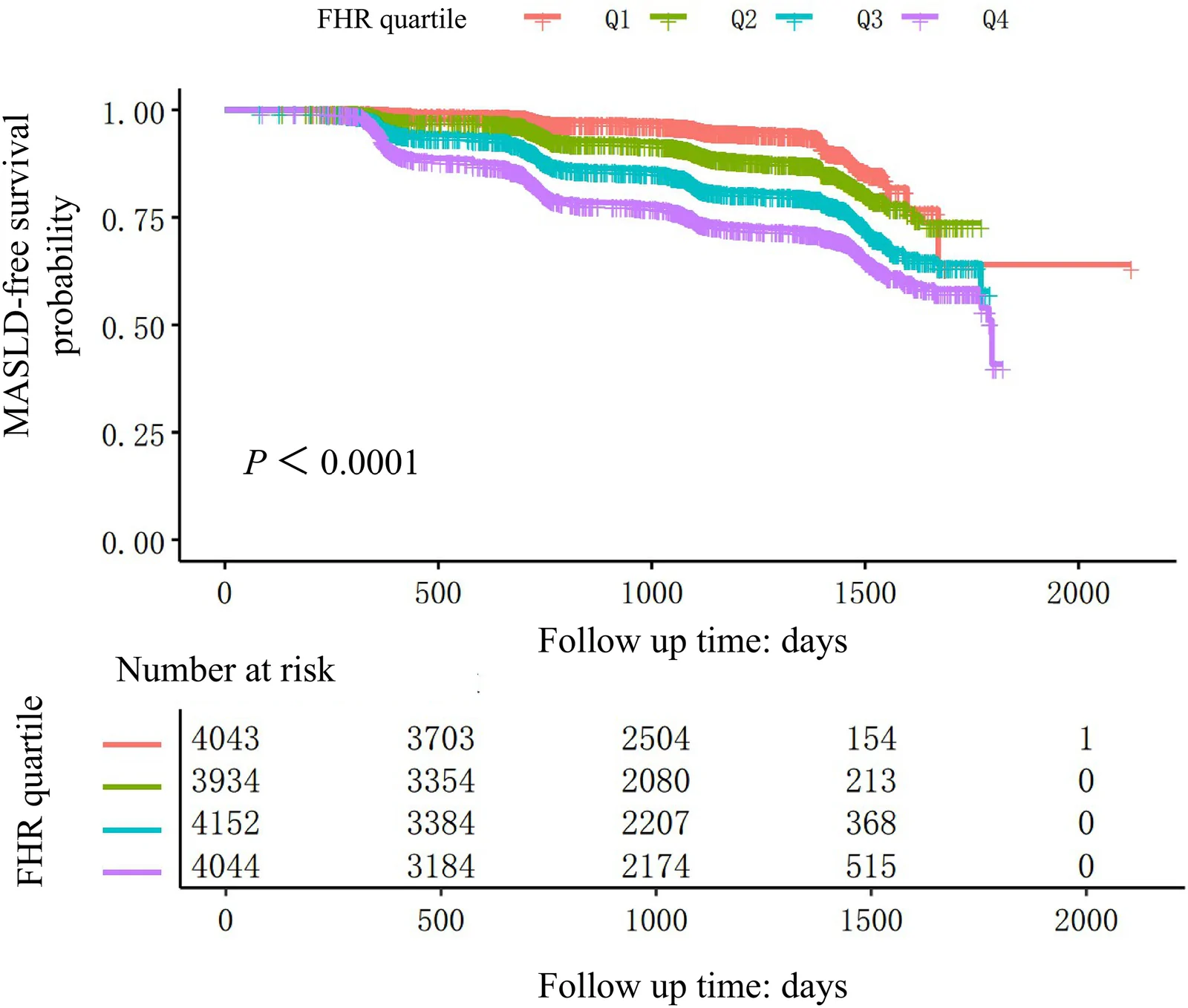
Kaplan–Meier curves of cumulative incidence of new onset MASLD stratified by FHR quartile. FHR = fasting plasma glucose to high-density lipoprotein cholesterol ratio, MASLD = metabolic dysfunction-associated steatotic liver disease.

### 3.4. Subgroup analyses and interaction testing

To assess the robustness of the association between FHR and the risk of MASLD, a series of prespecified subgroup analyses were conducted (Table 3). The positive association between FHR and MASLD risk remained consistent across various subgroups stratified by factors such as sex, age, blood pressure, liver enzymes (ALT, AST, and ALP), renal function (Cr), UA, and TC. No significant interactions were observed between these subgroups (*P* > .05). Notably, a significant interaction was observed between baseline TG levels (*P* for interaction = 0.0089). Although FHR was an independent risk factor for MASLD in both low-TG (<1.7 mmol/L) and high-TG (≥1.7 mmol/L) subgroups, the strength of the association differed. The association was stronger in the subgroup with high TG levels (≥1.7 mmol/L; Table 3).

**Table 3:** The effect size of FHR on MASLD in prespecified and exploratory subgroups in each subgroup.

FHR	N	HR (95% CI) *P*-value	*P* interaction
Sex			.2341
Female	7690	1.14 (1.07–1.22) **<**.0001	
Male	8483	1.25 (1.18–1.32) **<**.0001	
Age, yr			.2204
<30	3548	1.32 (1.19–1.46) **<**.0001	
≥30, <45	6480	1.17 (1.09–1.25) **<**.0001	
≥45, <60	3777	1.13 (1.04–1.23) .0038	
≥60	2368	1.27 (1.14–1.41) **<**.0001	
SBP, mm Hg			.3433
<140	14,090	1.22 (1.16–1.28) **<**.0001	
≥140	2083	1.20 (1.10–1.31) **<**.0001	
DBP, mm Hg			.4953
<90	15,024	1.22 (1.16–1.27) **<**.0001	
≥90	1149	1.23 (1.10–1.37) .0002	
GGT, U/L			.3792
<40	14,184	1.20 (1.14–1.27) **<**.0001	
≥40	1989	1.17 (1.08–1.26) .0001	
ALT, U/L			.6736
≤40	15,488	1.22 (1.17–1.28) **<**.0001	
>40	685	1.22 (1.06–1.40) .0057	
AST, U/L			.7102
≤40	15,841	1.22 (1.17–1.28) **<**.0001	
>40	332	1.12 (0.89–1.39) .3275	
ALP, U/L			.3813
<120	15,836	1.22 (1.17–1.27) **<**.0001	
≥120	337	1.17 (0.96–1.42) .1242	
TP, g/L			.5553
≤65	208	1.80 (1.03–3.13) .0390	
>65, ≤80	14,983	1.21 (1.15–1.26) **<**.0001	
>80	982	1.28 (1.07–1.53) .0064	
ALB, g/L			.3547
<40	760	1.07 (0.87–1.31) .5188	
≥40	15,413	1.23 (1.18–1.29) **<**.0001	
GLB, g/L			.2640
<35	15,046	1.21 (1.16–1.27) **<**.0001	
≥35	1127	1.33 (1.13–1.57) .0006	
CR, µmo/L			.9242
<104	14,760	1.20 (1.14–1.25) **<**.0001	
≥104	1413	1.29 (1.15–1.45) **<**.0001	
UA, mmol/L			.5845
<360	13,330	1.23 (1.17–1.30) **<**.0001	
≥360	2843	1.21 (1.12–1.30) **<**.0001	
TG, mmol/L			.0089
<1.7	13,090	1.16 (1.09–1.24) **<**.0001	
≥1.7	3083	1.18 (1.12–1.25) **<**.0001	
TC, mmol/L			.1489
<5.2	12,563	1.20 (1.15–1.26) **<**.0001	
≥5.2	3610	1.31 (1.22–1.40) **<**.0001	

ALB = albumin, ALP = alkaline phosphatase, ALT = alanine aminotransferase, AST = aspartate aminotransferase, BMI = body mass index, BUN = blood urea nitrogen, Cr = creatinine, DBP = diastolic blood pressure, FHR = fasting plasma glucose to high-density lipoprotein cholesterol ratio, FPG = fasting plasma glucose, GGT = gamma-glutamyl transferase, GLB = globulin, HDL-C = high-density lipoprotein cholesterol, LDL-C = low-density lipoprotein cholesterol, MASLD = Metabolic Dysfunction-Associated Steatotic Liver Disease, SBP = systolic blood pressure, TC = total cholesterol, TG = triglyceride, TP = total protein, UA = uric acid.

### 3.5. Assessment of the predictive value of FHR for MASLD

Receiver operating characteristic (ROC) curve analysis was used to evaluate the predictive value of FHR and its individual components (FPG and HDL-C) for incident MASLD (Fig. 4). The results showed that the area under the curve (AUC) for FHR was 0.701, which was superior to that of HDL-C alone (AUC: 0.667) or FPG alone (AUC: 0.645). This indicates that compared to its individual components, FHR has better predictive performance for the development of MASLD in the nonobese population.

**Figure 4.**
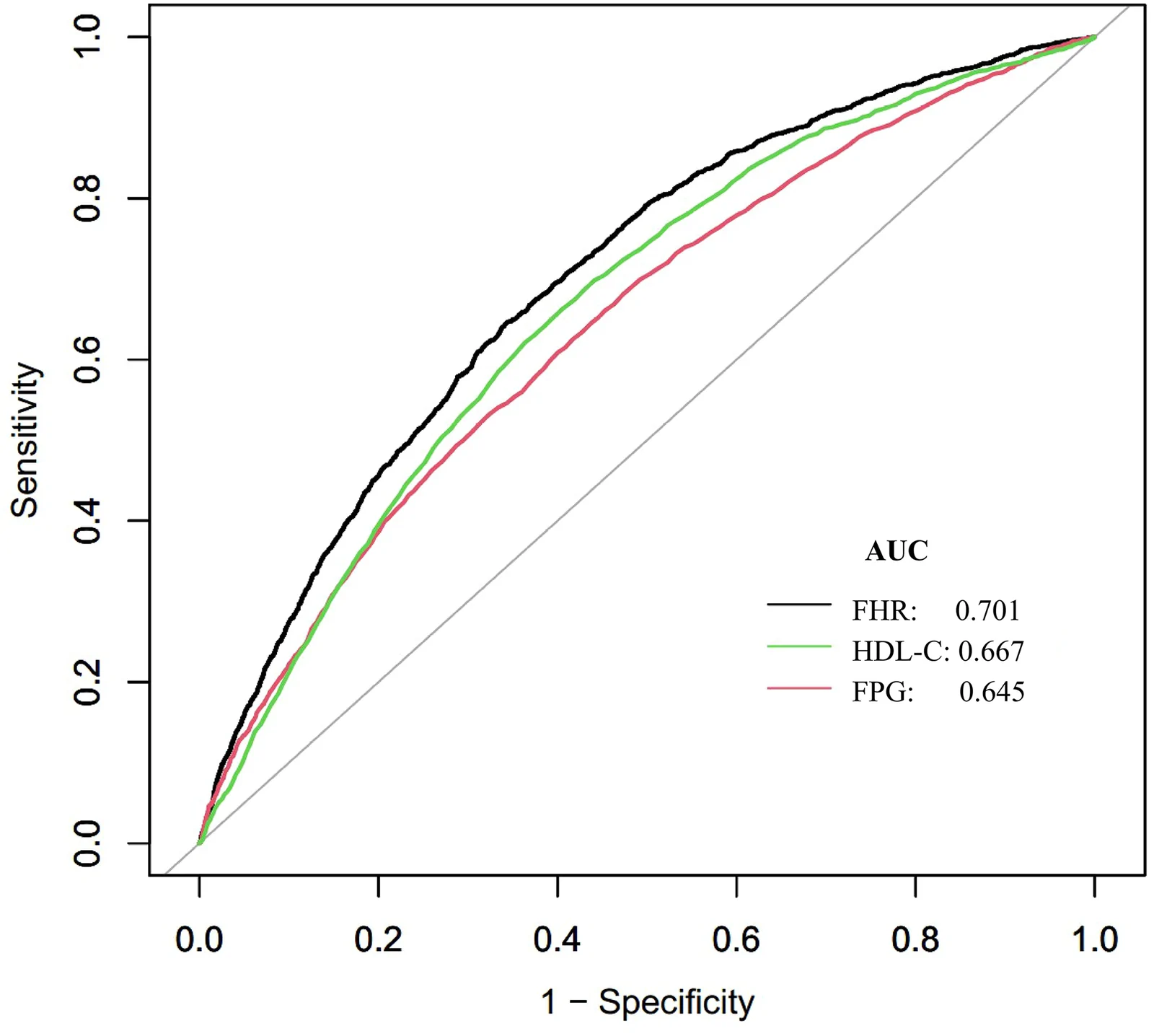
ROC curve for MASLD. MASLD = metabolic dysfunction-associated steatotic liver disease, ROC = receiver operating characteristic.

## 4. Discussion

In this retrospective cohort study conducted in China, we found that FHR was significantly associated with the risk of incident MASLD in nonobese adults. A clear dose–response relationship was observed, and the association remained robust after adjustment for multiple confounders. These findings suggest that FHR may serve as a useful indicator for identifying individuals at increased risk of MASLD.

Our findings extend previous evidence derived from cross-sectional studies. Several prior investigations have reported a significant association between FHR and MASLD or NAFLD in different populations. For instance, an analysis based on the National Health and Nutrition Examination Survey demonstrated a strong relationship between FHR and prevalent NAFLD in US adults,^[16]^ and similar findings have been reported in Japanese populations.^[17]^ However, due to the inherent limitations of cross-sectional designs, these studies were unable to establish the temporal sequence between exposure and outcome. In contrast, the prospective design of the present study allows for the observation that elevated baseline FHR precedes the development of MASLD, thereby providing additional longitudinal evidence supporting its potential predictive value.^[22]^

The observed association between FHR and MASLD is biologically plausible. FHR integrates 2 key metabolic components involved in MASLD pathogenesis: insulin resistance (IR)^[23,24]^ and dyslipidemia.^[25]^ Elevated fasting plasma glucose reflects impaired glucose metabolism and is closely linked to systemic IR,^[26,27]^ which promotes hepatic fat accumulation through increased free fatty acid flux to the liver,^[28,29]^ and enhanced de novo lipogenesis.^[30]^ Meanwhile, reduced HDL-C levels are associated with impaired reverse cholesterol transport and diminished anti-inflammatory and antioxidant functions.^[31,32]^ As a composite index, FHR may therefore capture the combined effects of glucotoxicity and lipotoxicity more effectively than individual markers, providing a more comprehensive reflection of metabolic dysfunction related to hepatic steatosis.^[17,33]^

Another important aspect of this study is its focus on the nonobese Chinese population. Previous large-scale studies, including those based on NHANES data, have primarily involved Western populations.^[16]^ Given known differences in genetic background, lifestyle factors, and metabolic characteristics between Asian and Western populations, particularly in the context of nonobese MASLD, direct extrapolation of findings may be limited.^[3]^ Our results provide population-specific evidence supporting the relevance of FHR in this subgroup and contribute to a better understanding of MASLD risk in nonobese individuals.^[34,35]^

We also observed that the optimal cutoff value of FHR for predicting MASLD in this study was 3.87 (AUC = 0.701; Table S3, Supplemental Digital Content 3), which is comparable to that reported in a Japanese population (3.89, AUC = 0.79),^[16]^ but lower than that observed in a US population (4.727, AUC = 0.731).^[17]^ These differences may reflect ethnic variations in metabolic profiles.^[36,37]^ It has been suggested that Asian populations tend to have higher visceral fat accumulation and greater susceptibility to insulin resistance at lower body mass index levels compared with Western populations.^[38]^ Such differences may contribute to variation in the threshold at which metabolic indices such as FHR become clinically relevant. Despite these differences in cutoff values, the predictive performance of FHR across studies remains within a comparable range, suggesting a degree of consistency in its utility across populations.

This study has several strengths. First, the large sample size provided greater statistical precision, while the longitudinal structure of this retrospective cohort allowed assessment of the association between baseline FHR and MASLD identified during subsequent follow-up. Second, the relatively long follow-up period provided an extended observation window for identifying MASLD cases. Third, the focus on nonobese individuals addresses an important but often under-recognized subgroup. In addition, we adjusted for a wide range of potential confounders and applied multiple imputation methods to handle missing data, thereby reducing the loss of information associated with complete-case analysis.

Several limitations should also be acknowledged. First, the retrospective observational design is susceptible to selection bias and information bias and does not establish causality. Second, hepatic steatosis was assessed using abdominal ultrasonography rather than histological or advanced imaging methods. The limited sensitivity of ultrasonography for mild steatosis may have led to misclassification, including undetected disease at baseline. Consequently, MASLD detected during follow-up may not reflect the true timing of disease onset. The temporal ordering of baseline FHR measurements and subsequent MASLD detection therefore does not establish that elevated FHR preceded the biological onset of MASLD, and reverse causation cannot be excluded. Third, FHR and other covariates were assessed only at baseline, and changes over time were not captured. Fourth, although multiple potential confounders were adjusted for, residual confounding cannot be excluded. In particular, information on smoking status and alcohol consumption below the original exclusion threshold was unavailable in the shared dataset, preventing adjustment for these exposures. Excluding participants who met the original alcohol-abuse criterion does not eliminate potential confounding by alcohol consumption among the retained participants. Other unmeasured factors, such as dietary habits, physical activity, and genetic variants (e.g., PNPLA3 polymorphisms), may also have influenced the observed association. Fifth, fasting insulin measurements were unavailable; therefore, the homeostasis model assessment of insulin resistance (HOMA-IR) could not be calculated. This precluded a direct comparison of FHR with an established surrogate measure of insulin resistance and limited the assessment of FHR as an indicator of insulin resistance. Sixth, this study is a secondary analysis of an existing public cohort, and findings from analyses involving overlapping participants should not be interpreted as independent replications. The FHR cutoff of 3.87 and the corresponding AUC of 0.701 were derived and evaluated in the same dataset, without split-sample, bootstrap-based internal, or external validation. Consequently, the apparent discriminatory performance may be optimistic, and the stability and generalizability of the cutoff remain uncertain. This cutoff should therefore be considered exploratory rather than a validated threshold for clinical screening or decision-making. Finally, the original eligibility restrictions and recruitment of participants from a specific region in China limit the generalizability of the findings. In particular, the exclusion of participants with LDL-C > 3.12 mmol/L restricts applicability to the broader nonobese adult population, while the exclusion of those with BMI ≥ 25 kg/m^2^ precludes direct extrapolation to adults above this BMI threshold. Further multicenter studies involving independent cohorts with broader eligibility criteria are needed to assess the reproducibility and generalizability of the present findings. Specifically, the association between baseline FHR and subsequent MASLD detection, together with the performance of the proposed cutoff, should be externally validated in nonobese populations, while applicability to obese populations should be evaluated separately. Until such evidence is available, the utility of FHR for MASLD risk assessment and screening remains uncertain.

In summary, our findings suggest that elevated FHR is associated with an increased risk of MASLD in nonobese adults and may have potential utility as an accessible marker for risk stratification. Further studies are needed to validate these findings in other populations and to explore how FHR may be incorporated into clinical or public health screening strategies. In addition, future prospective studies should investigate whether interventions targeting individuals with elevated FHR can reduce the risk of MASLD development.^[39]^

## 5. Conclusions

In this large retrospective cohort study, a higher baseline FHR was associated with an increased risk of incident MASLD in nonobese Chinese adults after adjustment for measured confounders. FHR may be a useful marker for risk stratification; however, its incremental predictive value and clinical utility require further evaluation in independent cohorts.

## Acknowledgments

We would like to express our gratitude to all those who helped us in the writing of this manuscript. We also thank anonymous reviewers for their valuable comments.

## Author contributions

**Conceptualization:** Xueyan Wu.

**Data curation:** Fang Yi, Xueyan Wu.

**Formal analysis:** Jian Luo.

**Investigation:** Zhijie Zhuang, Haifeng Zhou, Danfeng Gong, Liqun Nie.

**Methodology:** Danfeng Gong, Liqun Nie.

**Validation:** Fang Yi.

**Visualization:** Yingzhu Xiong, Haifeng Zhou.

**Software:** Jian Luo.

**Writing – original draft:** Jian Luo, Zhijie Zhuang, Yingzhu Xiong.

**Writing – review & editing:** Xueyan Wu.






