# Supplementary material for: Fasting glucose-to-HDL cholesterol ratio and risk of MASLD in nonobese Chinese adults: A retrospective cohort study
Source: Medicine (Baltimore). 2026 Nov 6;105(45):e51474. doi: 10.1097/MD.0000000000051474 (PMC13747442; doi:10.1097/MD.0000000000051474)
Supplement: Supplementary file 1 [file medi-105-e51474-s001.docx]

**Supplementary Table S1. Sensitivity analysis before missing-data imputation: Association between FHR and MASLD in different models.**

| **Exposure** | **MASLD, HR (95%CI)** | | | | | |  |  |
| --- | --- | --- | --- | --- | --- | --- | --- | --- |
|  | **Crude model** | **model 1** | **model 2** | **Model 3** | **Model 4** | **model 5** |  |  |
| **FHR** | **1.59 (1.54, 1.65) <0.0001** | **1.58 (1.53, 1.64) <0.0001** | **1.41 (1.35, 1.46) <0.0001** | **1.41 (1.35, 1.47) <0.0001** | **1.32 (1.26, 1.39) <0.0001** | **1.19 (1.13, 1.25) <0.0001** |  |  |
| **FHR quartile** | | | | | | |  |  |
| **Q1** | **1.0** | **1.0** | **1.0** | **1.0** | **1.0** | **1.0** |  |  |
| **Q2** | **1.94 (1.63, 2.30) <0.0001** | **1.93 (1.63, 2.30) <0.0001** | **1.76 (1.44, 2.14) <0.0001** | **1.69 (1.39, 2.06) <0.0001** | **1.54 (1.26, 1.88) <0.0001** | **1.35 (1.10, 1.65) 0.0037** |  |  |
| **Q3** | **3.40 (2.91, 3.98) <0.0001** | **3.38 (2.89, 3.95) <0.0001** | **2.94 (2.46, 3.52) <0.0001** | **2.78 (2.32, 3.33) <0.0001** | **2.32 (1.92, 2.81) <0.0001** | **1.85 (1.53, 2.24) <0.0001** |  |  |
| **Q4** | **4.96 (4.27, 5.76) <0.0001** | **4.89 (4.21, 5.69) <0.0001** | **3.77 (3.16, 4.48) <0.0001** | **3.63 (3.04, 4.33) <0.0001** | **2.95 (2.43, 3.58) <0.0001** | **2.08 (1.71, 2.53) <0.0001** |  |  |
| ***P* for trend** | **<0.0001** | **<0.0001** | **<0.0001** | **<0.0001** | **<0.0001** | **<0.0001** |  |  |

**Crude model adjusted for none;
model 1 adjusted for: SEX; AGE**

**model 2 adjusted for: SEX; AGE; TP; ALB; GLB; ALP; GGT; ALT; AST;**

**model 3 adjusted for: SEX; AGE; TP; ALB; GLB; ALP; GGT; ALT; AST; BUN; CR; UA  
model 4 adjusted for: SEX; AGE; TP; ALB; GLB; ALP; GGT; ALT; AST; BUN; CR; UA; TC1; TG; LDL
model 5 adjusted for: SEX; AGE; TP; ALB; GLB; ALP; GGT; ALT; AST; BUN; CR; UA; TC1; TG; LDL; SBP; DBP; BMI**
