# Supplementary material for: Fasting glucose-to-HDL cholesterol ratio and risk of MASLD in nonobese Chinese adults: A retrospective cohort study
Source: Medicine (Baltimore). 2026 Nov 6;105(45):e51474. doi: 10.1097/MD.0000000000051474 (PMC13747442; doi:10.1097/MD.0000000000051474)
Supplement: Supplementary file 2 [file medi-105-e51474-s002.docx]

**Supplementary Table S2. Threshold effect analysis of the association between FHR and MASLD in the overall population and stratified by sex.**

**For exposure: FHR**

| **Outcome:** | **MASLD** | **MASLD（Female）** | **MASLD（Male）** |
| --- | --- | --- | --- |
| **Model I** |  |  |  |
| **One-line effect** | **1.2 (1.2, 1.3) <0.001** | **1.1 (1.1, 1.2) <0.001** | **1.2 (1.2, 1.3) <0.001** |
| **Model II** |  |  |  |
| **Breakpoint (K)** | **3.5** | **3.5** | **4** |
| **Effect 1 (< K segment)** | **2.0 (1.7, 2.3) <0.001** | **1.9 (1.5, 2.5) <0.001** | **1.7 (1.4, 1.9) <0.001** |
| **Effect 2 (> K segment)** | **1.1 (1.1, 1.2) <0.001** | **1.0 (1.0, 1.1) 0.317** | **1.1 (1.0, 1.2) 0.031** |
| **Difference between Effect 2 and 1** | **0.6 (0.5, 0.7) <0.001** | **0.5 (0.4, 0.7) <0.001** | **0.7 (0.5, 0.8) <0.001** |
| **Log-likelihood ratio test** | **<0.001** | **<0.001** | **<0.001** |

**Outcome variable: MASLD**

**Exposure variable: FHR**

**Adjusted variables: SEX; AGE; TP; ALB; GLB; ALP; GGT; ALT; AST; BUN; CR; UA; TC1; TG; LDL; SBP; DBP; BMI**
