# Supplementary material for: Fasting glucose-to-HDL cholesterol ratio and risk of MASLD in nonobese Chinese adults: A retrospective cohort study
Source: Medicine (Baltimore). 2026 Nov 6;105(45):e51474. doi: 10.1097/MD.0000000000051474 (PMC13747442; doi:10.1097/MD.0000000000051474)
Supplement: Supplementary file 3 [file medi-105-e51474-s003.docx]

**Supplementary Table S3. ROC analysis of FHR, FBG, and HDL-C for discriminating MASLD.**

| **Test** | **ROC area(AUC)** | **95%CI low** | **95%CI upp** | **Best threshold** | **Specificity** | **Sensitivity** |
| --- | --- | --- | --- | --- | --- | --- |
| **FHR** | **0.701** | **0.690** | **0.713** | **3.87** | **0.658** | **0.650** |
| **FBG** | **0.645** | **0.633** | **0.657** | **5.10** | **0.599** | **0.609** |
| **HDL-C** | **0.667** | **0.655** | **0.678** | **1.34** | **0.637** | **0.621** |
